# Supplementary material for: Association between Galectin Levels and Neurodegenerative Diseases: Systematic Review and Meta-Analysis
Source: Biomolecules. 2022 Jul 31;12(8):1062. doi: 10.3390/biom12081062 (PMC9406080; doi:10.3390/biom12081062)
Supplement: Supplementary file 1 [file biomolecules-12-01062-s001.zip › biomolecules-1749140-supplementary.pdf]

Table S1. Newcastle–Ottawa quality assessments scale.

Studies	Selection				Comparability	Outcome			Scores
	Is the case definition adequate?	Representativeness of the cases	Selection of Controls	Definition of Controls	Comparability of cases and controls on the basis of the design or analysis	Ascertainment of exposure	Same method of ascertainment for cases and controls	Non-Response rate	
Ashraf, 2018	*	*	*	*	*	*	*	*	8
Basso, 2004	*	*	*	*	*	*	*	*	8
Boza-Serrano, 2019	*	*	*	*	*	*	*	*	8
Burman, 2016	*	*	*	*	*	*	*	*	8
Cengiz, 2019	*	*	*	*	*	*	*	*	8

**Marques, 2019**	*	*	*	*	*	*	*	*	**8**
**Stancic, 2011**	*	*	*	*	*	*	*	*	**8**
**Tao, 2020**	*	-	-	*	-	*	*	*	**5**
**Tian, 2016**	*	*	*	*	*	*	*	*	**8**
**Wang, 2015**	*	*	*	*	*	*	*	*	**8**
**Wang, 2019**	*	*	*	*	*	*	*	*	**8**
**Werner, 2008**	*	*	*	*	*	*	*	*	**8**
**Wu, 2021**	*	*	*	*	*	*	*	*	**8**
**Yan, 2016**	*	*	*	*	*	*	*	*	**8**

**Yazar, 2021**	*	*	*	*	*	*	*	*	**8**
**Zhou, 2010**	*	-	-	*	-	*	*	*	**5**
